# Drosophila balancer reengineering using polycistronic gRNA for CRISPR/Cas9 gene editing

**DOI:** 10.17912/sg7d-sd61

**Published:** 2018-11-13

**Authors:** Leif Benner, Brian Oliver

**Affiliations:** 1 Section of Developmental Genomics, Laboratory of Cellular and Developmental 10 Biology, National Institute of Diabetes and Digestive and Kidney Diseases, National Institutes of Health, Bethesda, MD, 20892.; 2 Department of Biology, Johns Hopkins University, Baltimore, MD, 21218.

**Figure 1.**
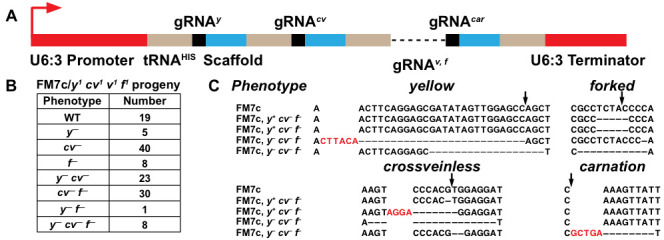
A) U6:3-tRNA^His^-gRNA*^y,cv,v,f,car^* cassette design. Red arrow represents transcription start site. B) Number of phenotypic mutants transmitted from *Act5c-*Cas9/*FM7c; U6:3-tRNA^His^-gRNA^y,cv,v,f,car^*/+ females. Genotypes of the progeny are *FM7c*/*y^1^ cv^1^ v^1^ f^1^*. C) Mutations induced at each locus of the respective phenotypes from B. Red letters represent insertions and dashes represent deletions. Black arrows show the Cas9 cleavage site.

## Description

A number of reagents have been generated for CRISPR/Cas9 genome editing in *Drosophila* (Gratz et al. 2014; Ren et al. 2013; Kondo and Ueda 2013; Port et al. 2014; Port and Bullock 2016). The ability to efficiently express multiple gRNAs from a single vector provides the advantage of creating multiple CRISPR/Cas9 alleles at a time. An elegant design has previously shown that gRNAs separated by tRNAs allow for genomic editing at multiple loci in *Drosophila* (Port and Bullock 2016). We generated a similar polycistronic gRNA cassette under the control of the *Drosophila* *snRNA:U6:96Ac* (U6:3) regulatory sequences. Individual gRNAs were separated by the *Drosophila* histidine tRNA and targeted the genes *yellow* (*y*), *crossveinless* (*cv*), *vermilion* (*v*), *forked* (*f*), and *carnation* (*car*) along the X chromosome (A). In theory, after expression of the gRNA cassette, the tRNAs should lead to the formation of secondary RNA structures and be recognized for cleavage by RNase enzymes P and Z (Frendewey et al. 1985; Dubrovsky et al. 2004), releasing the gRNAs for incorporation into Cas9. The gRNA cassette (U6:3-tRNA^HIS^-gRNA*^y,cv,v,f,car^*) was stably integrated into the *Drosophila* genome and crossed to a Cas9 line under the control of the Act5C promoter (Zhang, Koolhaas, and Schnorrer 2014). Females with the genotype Act5c-Cas9/FM7c; U6:3-tRNA^HIS^-gRNA*^y,cv,v,f,car^*/+ showed y^–^, cv^–^, and f^–^ phenotypes while females with the genotype *y^1^ w^1^/FM7c; U6:3-tRNA^HIS^-gRNA^y,cv,v,f,car^/+* were wild type. We then outcrossed *Act5c-Cas9/FM7c; U6:3-tRNA^HIS^-gRNA^y,cv,v,f,car^/+* females to a *y^1^ cv^1^ v^1^ f^1^* stock and scored germline transmission of editing at *y*, *cv*, and *f*, on the FM7c balancer chromosome. Germline transmission resulted in 14% of progeny not mutant at any locus, 40% were mutant at one locus, 40% were mutant at two loci, and 6% were mutant at three loci (B). We sequenced the targeted genomic sites of two phenotypically y^+^ cv^–^ f^–^ and two y^–^ cv^–^ f^–^ balancers to compare with the original FM7c chromosome. All flies contained out-of-frame indels at the loci consistent with their phenotypes. None of the flies had mutations at *v*, and one fly had an in-frame indel at *car* (C). We are unsure why these flies did not show a car^–^ phenotype, however, our sequencing results indicate that germline transmission of editing events did occur at the *car* locus. Our data suggests that gRNAs targeting *y*, *cv*, *f*, and *car* were cleaved from the cassette and loaded into Cas9 allowing for subsequent genomic editing. The ability to express multiple gRNAs from a single vector offers advantages over traditional gRNA vectors for *Drosophila* and will allow researchers to expand their experimental repertoire for CRISPR/Cas9 genome editing. Practically, polycistronic gRNA vectors can be used to quickly generate and expand new balancer genotypes, that would otherwise be difficult and time-consuming through traditional methods.

## Methods

U6:3-tRNA^HIS^-gRNA*^y,cv,v,f,car^* was synthesized by GenScript (Piscataway, NJ) and subcloned into pUASt-attB (Bischof et al. 2007) generating pU6:3-tRNA^HIS^-gRNA*^y,cv,v,f,car^*. pU6:3-tRNA^HIS^-gRNA*^y,cv,v,f,car^* was integrated into the *P{CaryP}attP40* landing site (Markstein et al. 2008) through phi-C31 mediated integration (Bischof et al. 2007). Embryo injections for transgenesis and transformant recovery was completed by BestGene (Chino Hills, CA). Sanger sequencing was completed by Genewiz (Plainfield, NJ). Flies were raised on ‘Fly Food B’ (LabExpress, Ann Arbor, MI) at 25°C. A y^–^ phenotype was scored by a reduction in pigmentation of the bristles for *y^31d^* on FM7c. All sequences are available upon request.

## Reagents

FM7c

FM7c, cv^WJB^ f^PDB^

y^1^ v^1^; P{CaryP}attP40

y^1^ M{vas-int.Dm}ZH-2A w^*^

y^1^ M{Act5C-Cas9.P.RFP-}ZH-2A w^1118^ DNAlig4^169^

y^1^ w^1^; P{y^+t7.7^ w^+mC^=U6:3-tRNA^HIS^-gRNA^y,cv,v,f,car^}attP40
